# Neural Crest-Derived Co-localization: A Case of Granular Cell Tumor Arising Beneath a Nevus Spilus

**DOI:** 10.7759/cureus.88966

**Published:** 2025-07-29

**Authors:** Brian A Moreno, Moises Lutwak, Daniel Lutwak, Stanley Skopit

**Affiliations:** 1 Dermatology, Lake Erie College of Osteopathic Medicine, Bradenton, USA; 2 Dermatology, Larkin Community Hospital, South Miami, USA

**Keywords:** academic dermatology, adult dermatology, clinical dermatology, complex dermatology, dermatology case report, dermatology diagnosis, dermatology screening, general dermatology, medical dermatology, skin disease

## Abstract

Granular cell tumors (GCTs) are rare neoplasms believed to originate from Schwann cells, exhibiting immunohistochemical and ultrastructural features consistent with neural crest derivation. Similarly, a nevus spilus (NS) is a pigmented skin lesion composed of lentiginous background macules with superimposed darker macules or papules, also thought to arise from neural crest-derived melanocytes. We report a unique case of a 27-year-old female who presented with a painful subcutaneous nodule on the left medial midback, overlaid by an NS. Surgical excision revealed a lesion consistent with a GCT based on histopathologic evaluation. To our knowledge, this is one of the few reported cases demonstrating spatial co-localization of a GCT and NS. This case raises the possibility of a shared embryologic origin contributing to the concurrent development of both lesions in the same anatomic location. Further research is needed to explore whether this reflects a coincidental overlap or an underlying developmental association between neural crest-derived cutaneous proliferations.

## Introduction

**Figure 1:**
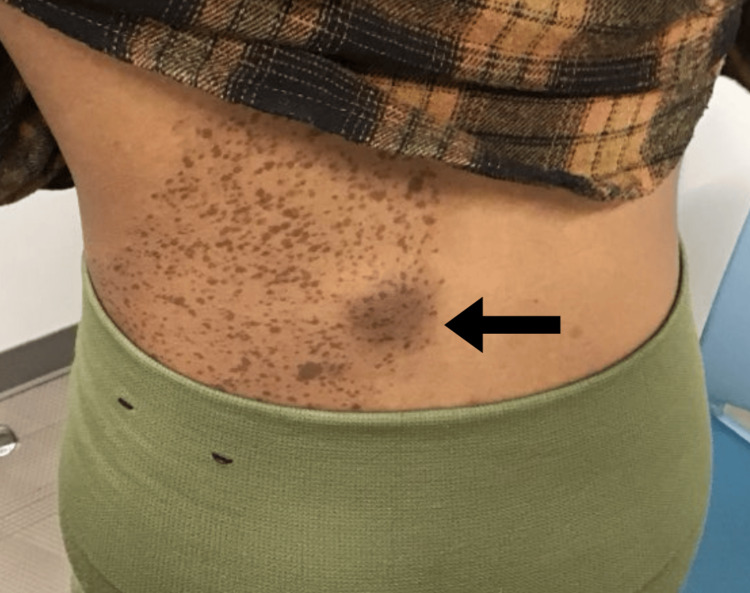
Clinical image showing the initial presentation of the subcutaneous nodule on the left medial midback. The overlying brown macular patch with darker speckled macules is consistent with a nevus spilus.

**Figure 2:**
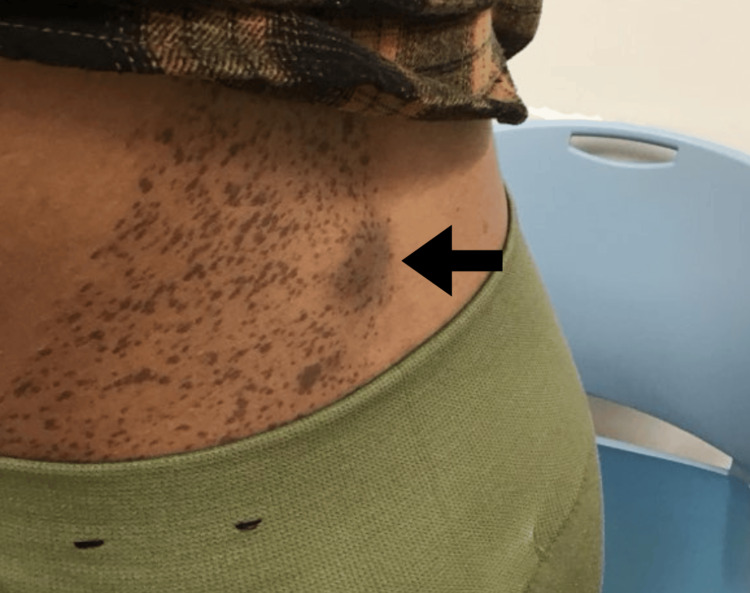
Magnified view of the nevus spilus overlying the symptomatic subcutaneous nodule. This pigmented lesion consists of a tan background with superimposed darker macules and papules.

Granular cell tumors (GCTs) are rare neoplasms of presumed neural crest origin, thought to arise from Schwann cells due to their consistent immunoreactivity for S-100 protein and ultrastructural features such as abundant lysosomes and myelin figures [1,2]. These tumors are typically benign and present as asymptomatic, slow-growing nodules most commonly located on the tongue, skin, and subcutaneous tissues, although they have been reported in nearly every organ system [2,3]. Histologically, GCTs are composed of polygonal cells with eosinophilic, granular cytoplasm and small, uniform nuclei, reflecting their abundant cytoplasmic lysosomes [3,4].

Nevus spilus (NS), also known as speckled lentiginous nevus, is a pigmented lesion characterized by a tan macular background with superimposed darker macules or papules, and is considered a hamartomatous malformation of melanocytes [5]. Like GCTs, NS is believed to be derived from neural crest cells, given its melanocytic origin and early embryological development [6]. While both entities are individually rare, their shared embryologic origin invites speculation about a potential developmental link when they occur concurrently.

To date, the co-localization of a GCT with an NS has not been widely documented. However, the presence of both lesions in the same anatomical region, each arising from distinct yet closely related neural crest-derived lineages, raises questions regarding possible embryological or microenvironmental factors contributing to their development. Furthermore, although the majority of GCTs follow a benign course, rare malignant variants have been reported, characterized by aggressive growth, local recurrence, and metastatic potential [7]. These considerations underscore the importance of histopathological evaluation in cases where a new lesion arises beneath or in association with a preexisting pigmented nevus.

## Case presentation

A 27-year-old female presented with a one-year history of an enlarging, painful subcutaneous nodule on the left medial midback (Figures 1, 2). The overlying brown macular lesion with darker speckled macules, visible in clinical images, was consistent with an NS based on its classic appearance and patient history. She denied any systemic symptoms and declined a full-body skin examination. Physical exam revealed a well-nourished, alert, and oriented patient in no acute distress. A focused cutaneous assessment noted a subcutaneous cyst with a prominent follicular pore on the left medial midback, along with xerosis on the distal pretibial regions bilaterally. Initial management included a seven-day course of oral doxycycline 100 mg twice daily and counseling on the benign nature of epidermal inclusion cysts. Given the persistent symptoms and concern for growth in a visually exposed area, the patient consented to excision.

**Figure 3:**
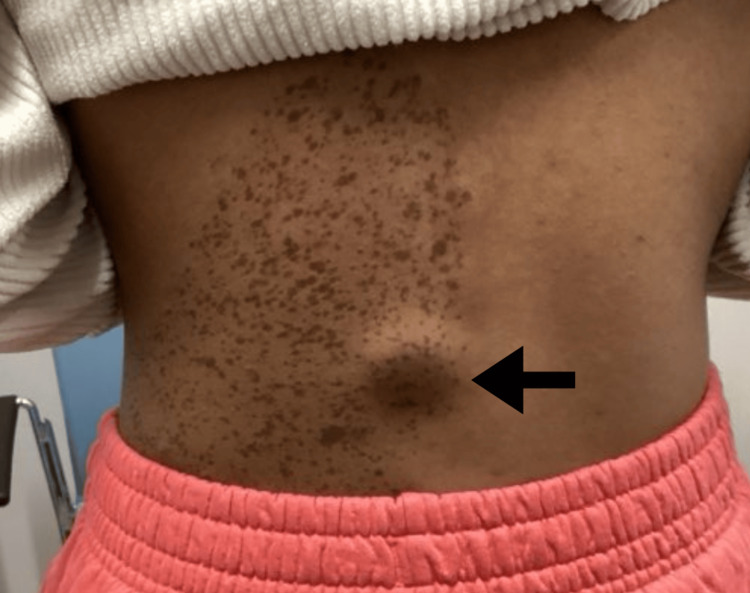
Clinical image showing a subcutaneous nodule on the left medial midback, located beneath a tan pigmented patch with darker speckled macules consistent with a nevus spilus.

**Figure 4:**
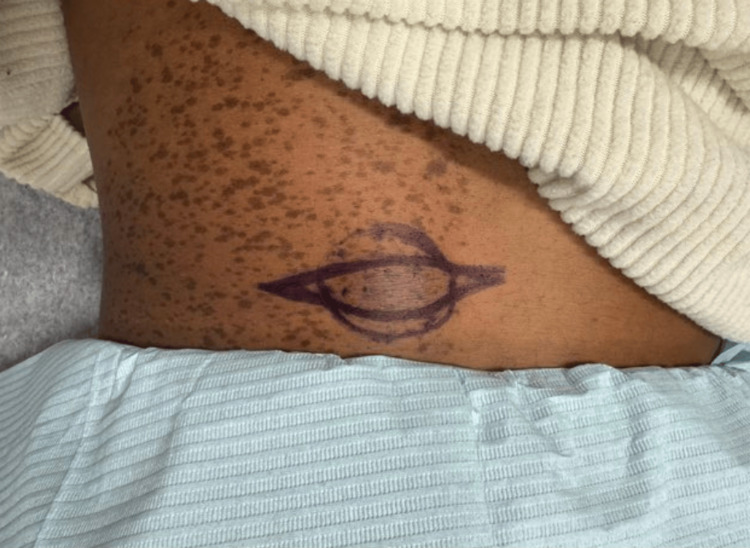
Fusiform excision of the subcutaneous nodule on the left medial midback with 0.2 cm clinical margins.

**Figure 5:**
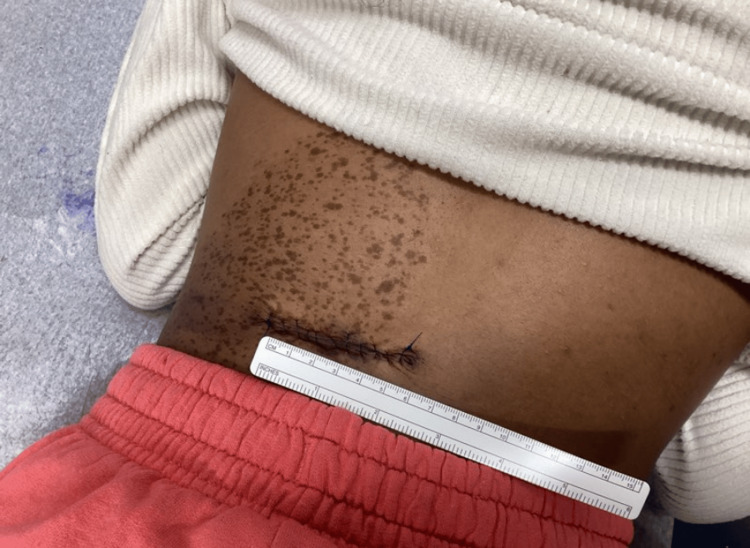
Final closure of a 6 cm surgical defect following complex repair with layered closure.

Two weeks later, the patient returned for a scheduled excision. At this visit, the lesion was documented as a subcutaneous nodule on the left medial midback that had grown appreciably since the previous visit (Figure 3). A fusiform excision with 0.2 cm margins was performed under local anesthesia (Figure 4). The total excised diameter measured 1.9 cm, and the final wound length was 6 cm following complex repair with layered closure (Figure 5). There were no intraoperative complications, and post-procedural care instructions were provided. No family history of similar lesions was reported, and laboratory or genetic testing was not performed. The excised specimen was sent for histopathological analysis.

At the two-week follow-up visit, sutures were removed without issue, and the surgical site was noted to be well-healed. Histopathologic analysis revealed features consistent with a benign GCT. While the histopathology supported this diagnosis, no ancillary genetic or molecular studies were performed to further characterize the lesion. The patient was counseled on the benign nature of the tumor and advised to monitor for any changes in size, shape, or color at the surgical site. Scar management recommendations were provided, and no recurrence or complications were noted at that time.

## Discussion

GCTs are rare neoplasms of Schwann cell origin, supported by immunohistochemical staining for S100 protein and electron microscopy findings of abundant lysosomes and granular cytoplasm [2,6]. Although most GCTs are benign, malignant variants account for fewer than 2% of cases and are associated with poor outcomes due to aggressive local behavior and metastatic potential [6]. GCTs commonly arise in the skin, tongue, and subcutaneous tissues, but can present anywhere in the body [1,2,6].

In this case, the GCT arose directly beneath an NS, a pigmented lesion composed of lentiginous background macules with superimposed nevi, including junctional, compound, or intradermal types [4,8]. While the co-occurrence of these two lesions has not been previously documented to our knowledge, both entities are derived from neural crest progenitors, raising the possibility of a shared embryologic origin or local microenvironmental influence [1,3,7]. The presence of a GCT beneath an NS may represent a coincidental finding; however, neural crest cells are known to retain plasticity and can respond to common signaling cues, which may predispose a shared anatomical site to multiple proliferative processes [3,9]. In addition, recent studies have described concurrent neural crest-derived proliferations in the same anatomical region, such as the development of GCTs adjacent to congenital nevi, lending further plausibility to this hypothesis [10].

Clinically, GCTs may resemble benign lesions such as epidermal inclusion cysts or dermatofibromas, particularly when located in the dermis or subcutis [2,5]. In our patient, the lesion was initially suspected to be an inclusion cyst based on clinical presentation and was treated empirically with oral doxycycline. However, histopathologic evaluation following surgical excision demonstrated findings consistent with a GCT. This case illustrates the diagnostic challenges posed by GCTs and emphasizes the importance of biopsy in the evaluation of enlarging or symptomatic lesions. Although histopathologic features supported the diagnosis, the lack of genetic or laboratory evaluation represents a limitation in fully characterizing the lesion.

Histologically, GCTs demonstrate large polygonal cells with eosinophilic, granular cytoplasm and small, uniform nuclei. These tumors stain strongly for S100 and CD68, consistent with Schwannian and lysosomal differentiation [1,2]. NS, by contrast, typically shows an underlying lentiginous pattern with overlying melanocytic proliferations, and is sometimes associated with mosaic RAS pathway mutations [4,8]. Moreover, shared mutations in RAS-MAPK signaling components have been implicated in both GCTs and melanocytic lesions, providing a potential molecular link [11].

The exact pathogenesis underlying the simultaneous occurrence of GCT and NS is not well understood. Segmental mosaicism or somatic postzygotic mutations affecting neural crest derivatives may account for the development of dual lesions at the same site [7,8]. Studies have also shown that neural crest cells exhibit lineage plasticity influenced by local signaling gradients, which may promote divergent but co-localized phenotypic outcomes [12]. Further genetic and molecular studies are needed to evaluate whether such associations are coincidental or reflective of shared pathophysiologic mechanisms.

Complete surgical excision is the preferred treatment for benign GCTs, and recurrence is rare with negative margins. Long-term follow-up is still advised due to the potential, albeit low, for recurrence or malignant transformation [6]. This case highlights a unique presentation involving two distinct yet embryologically related lesions and adds to the growing literature on neural crest-derived skin pathologies.

## Conclusions

This case highlights a rare clinical scenario in which a GCT, a Schwann cell-derived neoplasm, developed beneath an NS, a melanocytic lesion also originating from neural crest cells. While the co-occurrence may be incidental, the shared embryologic lineage of both lesions invites consideration of a possible developmental or molecular link. Clinicians should maintain a high index of suspicion for alternative diagnoses when evaluating persistent or symptomatic dermal nodules, even in the presence of seemingly benign overlying pigmentation. Surgical excision with histopathologic evaluation remains essential for diagnosis and definitive management. Further research is warranted to explore potential associations between neural crest-derived lesions and to better understand the mechanisms driving their co-localization.
